# Basic and clinical significance of IGF-I-induced signatures in cancer

**DOI:** 10.1186/1741-7015-8-2

**Published:** 2010-01-05

**Authors:** Haim Werner, Ilan Bruchim

**Affiliations:** 1 Department of Human Molecular Genetics and Biochemistry, Sackler School of Medicine, Tel Aviv University, Tel Aviv 69978, Israel; 2 Gynecologic Oncology Unit, Department of Obstetrics and Gynecology, Meir Medical Center, Kfar Saba 44281, Israel

## Abstract

The insulin-like growth factor (IGF) system mediates growth, differentiation and developmental processes; it is also involved in various metabolic activities. Deregulation of IGF system expression and action is linked to diverse pathologies, ranging from growth deficits to cancer development. Targeting of the IGF axis emerged in recent years as a promising therapeutic approach in cancer and other medical conditions. Rational use of IGF-I-induced gene signatures may help to identify patients who might benefit from IGF axis-directed therapeutic modalities. In the accompanying research article in *BMC Medicine*, Rajski *et al*. show that IGF-I-induced gene expression in primary breast and lung fibroblasts accurately predict outcomes in breast and lung cancer patients.

See the associated research paper by Rajski et al: http://www.biomedcentral.com/1741-7015/8/1

## Introduction

The vast amount of information that has been generated in recent years, following the elucidation of the human genome, is changing our current views on most biological processes. Genomic and proteomic approaches, as well as other high-throughput platforms, are having a huge impact on our understanding of both basic and clinical questions. This 'gain-of-knowledge' is particularly manifest in the field of cancer research, where physiological and pathological processes are now amenable for integrative examination at multiple levels of analysis. The insulin-like growth factors (IGFs) are a collection of cellular and secreted factors with vital roles in cell biology. The IGF system consists of two ligands (IGF-I and IGF-II), two receptors [IGF-IR and IGF-II/mannose 6-phosphate receptor (M6P-R)] and at least six IGF-binding proteins (IGFBPs) [1-3]. In addition, the IGF network includes a series of IGFBP-related proteins and IGFBP proteases which are responsible for cleaving IGFBPs and, thus, releasing the free ligands. The IGF-IR, which mediates most of the biological actions of both IGF-I and IGF-II, is linked to the *ras-raf*-MAPK and PI3K-Akt signalling cascades [4,5].

The proliferative and antiapoptotic actions of IGF-I have been well characterized [6-8]. Although IGF-I, *per se*, is usually regarded as non-genotoxic (that is, it cannot induce mutations or cause transformation), once a malignant event has occurred, cell survival of already transformed cells depends to a large extent on IGF-I action. Breast tumours express all of the components of the IGF system, including ligands, receptors and IGFBPs [9]. Serum IGF-I stimulates breast cancer proliferation in an endocrine fashion. In addition, stromal cells adjacent to the malignant cells also produce IGF-I which acts in a paracrine manner. Breast cancer cells, themselves, produce mainly IGF-II. The importance of IGF action in cancer biology is supported by epidemiological studies showing a correlation between circulating IGF-I and cancer incidence. In a prospective nested control study (the Nurse's Health Study, published in 1998) the relative risk of breast cancer in premenopausal women was shown to be 4.6 in the upper tertile of IGF-I values in comparison to women in the lower tertile [10]. These seminal studies have been replicated over the last 10 years by numerous groups worldwide. Systematic reviews of the literature and meta analyses conclude, for the most part, that high circulating IGF-I concentrations are associated with an increased risk of premenopausal breast cancer, although the relative risks were substantially lower than those reported in early studies [11].

In recent years, the IGF system emerged as a promising interventional target in cancer therapy and major translational efforts are currently aimed at identifying and optimizing therapeutic tools directed against this growth factor system [12-14]. It is, therefore, crucial to identify IGF-I-induced genes that can predict clinical outcome in breast and other types of cancer.

## Discussion

This month in *BMC Medicine*, a paper by Rajski *et al*. [15] deals with the potential role of IGF-I-induced genes in primary breast and lung fibroblasts as predictors of outcome in breast and lung cancer patients. This report provides a comprehensive, yet highly focused, analysis of the potential clinical significance of IGF-I-induced signatures. Specifically, the authors used an *ex vivo *culture model and measured gene expression changes after IGF-I stimulation with cDNA microarrays. Using a series of bioinformatic tools (GO TermFinder, TreeView softwares, and others) they analysed the *in vitro *microarray data and compared it with published expression datasets on human cancer biopsies.

Characterization of IGF-I-induced genes in CCL-171 human primary fibroblasts detected an increase (greater than 1.5-fold change) in the expression levels of 370 genes. This fibroblast derived IGF-I signature was enriched for genes involved in biological processes such as mitotic cell cycle, angiogenesis, p53 pathway, integrin and Wnt signalling. IGF-I stimulation of MCF-7 breast cancer cells induced expression of genes involved in tumour biology (including the vascular endothelial growth factor) as well as genes involved in protein metabolism. In contrast to the upregulation of genes involved in proliferation observed in CCL-171 fibroblasts, the gene expression pattern in MCF-7 cells was not associated with cell cycle or proliferation.

In order to test the hypothesis that mesenchymal stromal cells and malignant epithelial cells exhibit distinct gene expression changes in response to IGF-I stimulation, the gene expression profiles of CCL-171 and MCF-7 cells, with and without IGF-I stimulation, were compared. Hierarchical clustering of the genes indicate that, when epithelial and mesenchymal cells are exposed to IGF-I, they show some concordant and some discordant gene expression changes. Genes that are upregulated in CCL-171 cells and downregulated in MCF-7 cells belong mainly to the Wnt and TGF-β signalling families and nucleic acid binding proteins. Genes that are upregulated in MCF-7 and downregulated in CCL-171 cells are mainly involved in protein metabolism and modification, as well as nucleoside, nucleotide and nucleic acid metabolism.

Carcinoma associated fibroblasts (CAF) and normal fibroblasts were obtained from three breast cancer patients, cultured and separated with magnetic beads targeting fibroblast-specific antigens. Both CAF and normal fibroblasts were then treated with IGF-I and gene expression profiles were generated. Two hundred and eight genes were shown to be upregulated by IGF-I and they were used to create the breast fibroblast derived IGF-I signature. The genes upregulated by IGF-I in primary fibroblasts share similar features to those elevated in CCL-171 fibroblast cultures. Contrary to the authors' expectations, they did not find any significant difference in the response to IGF-I between CAF and normal fibroblasts.

In order to verify the relevance of their *in vitro *data, the authors checked the expression of the breast fibroblast derived IGF-I signature in microarray data of early stage breast cancer biopsies from 295 patients. Interestingly, patients with a high expression level of this signature had a higher risk of developing metastases than patients with low expression levels. In addition, the overall survival rate was lower for patients with upregulated breast fibroblast derived IGF-I signature. The authors also show that the breast fibroblast derived IGF-I signature could segregate oestrogen receptor (ER) positive patients into two groups with significantly different clinical outcomes. Interestingly, patients with upregulation of this signature and ER negative status had the worse outcomes. The clinical correlation of the IGF-I signature should be further investigated, since it might have an important clinical application in treatment tailoring of breast cancer patients.

Finally, the breast fibroblast derived IGF-I signature was also positively correlated to previously published gene expression signatures, including the wound signature which was created based on the response of fibroblasts to serum stimulation [16]. In addition, the signature was inversely correlated to the good-risk 70-genes signature, which was created in order to predict freedom from metastasis [17]. The fibroblast derived IGF-I signature was also a prognostic factor in lung cancer.

## Conclusions

The study by Rajski *et al*. highlights the impact of novel profiling technologies on our ability to identify responders to specific targeted treatments. The IGF-I-induced signatures in primary breast and lung fibroblasts are similar to each other and also to previously published signatures, suggesting that these findings can be generalized to additional types of tumours. As indicated by the authors, the consistent response of fibroblasts to IGF-I might explain the worse outcome of patients with elevated circulating IGF-I values. Finally, and given the inconsistent correlation between IGF-IR expression and patient outcome in breast cancer [18], the IGF-I-induced signature might be of relevance for selecting patients who might benefit from IGF-IR blocking therapies.

## Abbreviations

CAF: carcinoma associated fibroblast; ER: oestrogen receptor; IGF: insulin-like growth factor.

## Competing interests

The authors declare that they have no competing interests.

## Authors' contributions

Both authors contributed equally to the writing of this article.

## Pre-publication history

The pre-publication history for this paper can be accessed here:

http://www.biomedcentral.com/1741-7015/8/2/prepub
